# The Impact of Age, Gender, Temporality, and Geographical Region on the Prevalence of Obesity and Overweight in Saudi Arabia: Scope of Evidence

**DOI:** 10.3390/healthcare11081143

**Published:** 2023-04-15

**Authors:** Hayfaa Wahabi, Amel A. Fayed, Zeinab Shata, Samia Esmaeil, Rasmieh Alzeidan, Elshazaly Saeed, Yasser Amer, Maher Titi, Khawater Bahkali, Layal Hneiny

**Affiliations:** 1 Research Chair for Evidence-Based Health Care and Knowledge Translation, King Saud University, Riyadh 11362, Saudi Arabia; hwahabi@ksu.edu.sa (H.W.); sesmaeil@ksu.edu.sa (S.E.); yamer@ksu.edu.sa (Y.A.); mtiti@ksu.edu.sa (M.T.); 2 Department of Family and Community Medicine, College of Medicine, King Saud University Medical City, Riyadh 12372, Saudi Arabia; 3 Department of Clinical Sciences, College of Medicine, Princess Nourah Bint Abdulrahman University, Riyadh 84428, Saudi Arabia; 4 High Institute of Public Health, Alexandria University, Alexandria 21544, Egypt; zeinab.shata@yahoo.com; 5 College of Medicine, Cardiac Sciences Department, King Saud University, Riyadh 11362, Saudi Arabia; ras_zeidan@hotmail.com; 6 College of Medicine, King Saud University, Riyadh 11362, Saudi Arabia; ehamed@ksu.edu.sa; 7 Pediatric Department, King Saud University, Riyadh 11362, Saudi Arabia; 8 Prince Abdullah Bin Khaled Coeliac Disease Research Chair, Pediatric Department, King Saud University, Riyadh 11362, Saudi Arabia; 9 Corporate Quality Management Department, King Saud University Medical City, Riyadh 11362, Saudi Arabia; 10 Department of Infectious Disease Epidemiology, London School of Hygiene and Tropical Medicine, London WC1E 7HT, UK; khawater_333@yahoo.com; 11 Department of Public Health Intelligence, Public Health Authority, Riyadh 11614, Saudi Arabia; 12 Wegner Health Sciences Library, University of South Dakota, Sioux Falls, SD 57069, USA; layal.hneiny@usd.edu

**Keywords:** Saudi Arabia, obesity, overweight, pooled prevalence

## Abstract

Objectives: The objectives of this scoping review are to estimate the prevalence of obesity and overweight in the Saudi community and in different age groups, genders, and geographical location, in addition to the change in prevalence over time. Methods: This scoping review of evidence was conducted in accordance with the Joanna Briggs Institute methodology for scoping reviews and was reported following the Preferred Reporting Items for Systematic reviews and Meta-Analyses extension for Scoping Reviews (PRISMA-ScR) guidelines. The population of this review was categorized into four age groups: young adults (18–25 years), adults (26–45), (mid-life adults) (46–60) and old people (60+). Each group was then categorized by gender into males and females. We included studies of adults aged 18 years and above. The pooled prevalence of obesity and overweight of the population, based on BMI, was estimated after stratification based on the age, gender, and geographical area. In addition, the change in the prevalence of obesity/overweight over time from 2011 to 2021 was investigated from the pooled data. The Metaprop program in Stata was used for statistical analysis. Results: A total of 39 studies with 640,952 participants were included in this review. The pooled prevalence of obesity and overweight in the age group of ≤25 years old, including both genders, was 30%. However, it was higher in young males (40%) compared to young females (25%). The prevalence of obesity and overweight among young adults has dropped by over 40% between 2012 and 2021. The overall pooled prevalence rate of obesity and overweight in the age groups >25 years old (adults, mid-life, and old people), including both genders, was 66%, with similar prevalence among males (68%) and females (71%). In addition, a similar prevalence was observed among both adult and old people (62% and 65%, respectively), but was higher in the mid-life group (76%). Furthermore, mid-life women had the highest prevalence among all groups (87%), compared to 77% among males in the same age group. The same difference in prevalence between the gender persisted in older females compared to older males (79% vs. 65%, respectively). There is a noticeable drop in the pooled prevalence of overweight and obesity among adults > 25 years old of over 28% between 2011 and 2021. There was no difference in the prevalence of obesity/overweight by geographical region. Conclusions: Despite the noticeable drop in the prevalence of obesity in the Saudi community, the prevalence of high BMI is high in Saudi Arabia irrespective of age, gender, or geographical location. Mid-life women have the highest prevalence of high BMI, which makes them the focus of a tailored strategy for intervention. Further research is needed to investigate which are the most effective interventions to address obesity in the country.

## 1. Introduction

Obesity has emerged as a global public health problem. The prevalence of obesity has increased significantly to a pandemic level during the last 50 years [1]. Recent reports showed that over 670 million adults worldwide were obese, in addition to 1.3 billion who were considered overweight in 2016 [1,2].

The prevalence of obesity and overweight in the Middle East was 21% and 33%, respectively, as estimated by a recently published systematic review [3]. Al-Quwaidhi et al. reported that the prevalence of obesity among adults in Saudi Arabia had increased from 22% in 1990 to 36% in 2005. The same study projected that there will be a considerable increase in obesity prevalence both in adult men and women by the year 2022 [4].

Similar to other high-income countries, 83% of the Saudi population lives in cities [5]. Rapid urbanization has had a negative impact on the prevalence of obesity and overweight in the country mainly due to limited physical activity with heavy traffic, and a lack of sport and gymnasium facilities and of sidewalks [6]. Many studies have reported a significant association between overweight and obesity and physical inactivity in Saudi Arabia [7,8,9]. Furthermore, there is a noticeable change in the pattern of diet in the Saudi community, with minimal consumption of fruits, vegetables, and fiber, and a high intake of sugar, saturated fat, and salt [10]. Published studies have demonstrated a significant association between overweight and obesity and unhealthy diet in the Saudi population [9,11,12]. In addition, socioeconomic determinants play a considerable role in the progressive increase in the prevalence of obesity in Saudi Arabia [5]. Apart from genetic predisposition, family size was found to be inversely associated with obesity in Saudi adolescents, along with parents’ level of education and employment [7].

Analysis of the recent Global Burden of Disease 2019 showed that a high BMI is one of the main risk factors for premature mortality and morbidity worldwide, including in the Middle East and Saudi Arabia [13]. High BMI is strongly linked to cardiovascular diseases, a leading cause of morbidity and preterm death both globally and in Saudi Arabia [14,15].

Obesity is a modifiable risk factor, which has the potential to be reversed or prevented. However, the prevalence of overweight and obesity in the Saudi Arabian population remains high, indicating ineffectiveness or lack of preventive measures and interventions [16,17]. Recently, many national programs and interventions have been implemented to reduce the prevalence of high BMI, however, the effectiveness of these programs has yet to be proven [18].

Different sectors of the community will respond to different types of interventions which are tailored according to the demographic characteristics of that group of the population, including gender and age [19,20]. Hence, the main objectives of this scoping review are to estimate the prevalence of obesity and overweight in the Saudi community among different age groups, genders, and geographical locations. This information will contribute to the framing of this health problem in its real perspective, so policy makers can set policies for proper allocation of resources to contain a long-standing and growing health problem.

## 2. Materials and Methods

### 2.1. Protocol and Registration

This scoping review was conducted in accordance with the Joanna Briggs Institute (JBI) methodology for scoping reviews [21]. The Preferred Reporting Items for Systematic reviews and Meta-Analyses extension for Scoping Reviews (PRISMA-ScR) guidelines were followed for reporting [22]. The scoping review protocol is registered in The Open Science Framework (OSF) [23].

### 2.2. This Scoping Review Addresses the following Research Questions

1. What is the prevalence rate of overweight and obesity among adults in the Saudi Arabian community in general and among subgroups of the population?
  - What is the overall prevalence of obesity and overweight in the Saudi Arabian community?
  - What is the distribution of obesity and overweight according to age?
  - What is the distribution of obesity and overweight according to gender?
  - What is the distribution of obesity and overweight according to geographical regions?
  - What is the temporality of obesity and overweight over the last 10 years?
2. What are the research gaps in the published literature on the prevalence of obesity and overweight in Saudi Arabia?

### 2.3. Information Sources and Search

The search strategy aimed to locate published studies. A medical librarian conducted an electronic literature search in six databases—MEDLINE (Ovid), Embase (Ovid), PyscINFO (Ovid), Cochrane CENTRAL (Ovid), Cochrane Database of Systematic Reviews (Ovid), and CINAHL (Ebsco)—from January 2011 till December 2021. A search strategy including all keywords and terms was adapted for each included database (Supplementary Materials). The reference lists of all included articles were screened for additional studies. There was no language limitation. The search keywords were: overweight, obesity, prevalence, incidence, obesity in adults, obesity in adolescence, obesity in pregnancy, obesity in geriatrics, cohort studies, cross sectional studies, systematic review, scoping review, and review.

### 2.4. Selection of Sources of Evidence

Once the search of the six databases was completed, the medical librarian uploaded the search results into EndNote reference manager and removed the duplicates. Two authors independently conducted title/abstract and full-text article screenings. Reasons for exclusion of articles that did not meet the inclusion criteria were reported. Any discrepancies regarding selection of included articles were reviewed by a third reviewer or resolved by discussion with additional reviewers.

### 2.5. Eligibility (Inclusion) Criteria

- The population was limited to the Saudi Arabian community including both male and female adults, in addition to a subgroup of participants including pregnant and postpartum women and geriatrics;
- Studies of the adult population (18 years and above). However, studies which included a sample starting from a minimum age of 15 years in which this age group could not be separated from adults were also included in the analysis;
- Populations in the community, educational institutions, or health institutions (e.g., hospital, health center);
- All quantitative observational research designs (descriptive, cohort, cross-sectional, case, and case series) and surveys with the primary objective of quantifying the prevalence of obesity and overweight;
- Obesity and overweight were reported as BMI;
- Published data were collected between January 2011 and December 2021.

### 2.6. Exclusion Criteria

- Studies on populations with conditions that may not be generalizable to the general population (e.g., chronic kidney disease, cancer);
- Studies on children and adolescents < 15 years old;
- Abstracts, conference proceedings, and grey literature.

### 2.7. Data Charting Process

Prevalence of obesity and overweight was reported for the total population and each subgroup from the included articles. However, when the age distribution reported in the article differed from the age distribution adopted in this review, re-calculation of the prevalence was performed for each age subgroup. Data was exported from EndNote to Google Sheets. Reviewers manually extracted data using the same sheet. Charting was done independently by two reviewers. Any disagreements between the reviewers were resolved by discussion with other reviewers.

### 2.8. Data Items

For the included studies, the following information was extracted into Google Sheets: study ID (author, publication year), study design, setting (hospital, school, etc.), geographical region and city, population type (students, workers, etc.), sample size, age range of the population (mean ± SD), and prevalence of obesity and overweight as percentages of the study population and gender.

### 2.9. Outcome Measures

For the analysis in this review, we combined overweight and obesity as high BMI due to the similar effect as a risk factor for other non-communicable diseases. On the basis of BMI, subjects were classified as either normal weight (<25 kg/m^2^), overweight (25–29.9 kg/m^2^), or obese (≥30 kg/m^2^) [24].

### 2.10. Data Synthesis

The extracted data was presented in tabular, forest plot, and map forms. In addition, a descriptive summary accompanied the tabulated and charted results to describe how the results relate to the research questions of this scoping review. To answer the research questions, we performed meta-analysis of proportions of overweight and obesity to pool the prevalence among different age groups and genders, trends over time, and across geographical regions. We used Metaprop program in Stata (StataCorp. 2019. Stata Statistical Software: Release 16.1 College Station, TX, USA: StataCorp LLC) that facilitates the pooling of proportions to report a summary of prevalence at different levels. The population of this review was categorized into four age groups: young adults (18–25 years), adults (26–45), mid-life adults (46–60), and old people (60+). Each group was then categorized by gender into males and females. We included studies of adults’ population 18 years and above. Studies which included a population with a minimum age of 15 years in which this age group could not be separated from adults were included in the analysis. In addition, each group of studies from the same geographical region (there are five geographical regions in Saudi Arabia), were analyzed together.

## 3. Results

**Figure 1:**
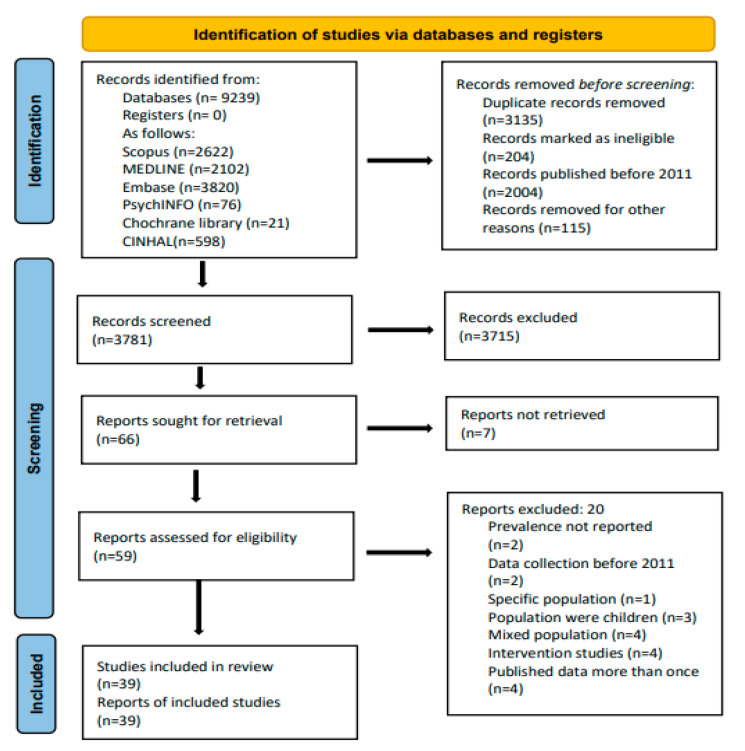
Prisma Flow Chart.

The published literature search identified a total of 9293 publications (Figure 1). After removal of duplicates (n = 3135), articles published before 2011 (2004), ineligible articles identified by automation tools (n = 204), and those excluded for other reasons (n = 115), a total of 3781 records remained. Screening by title and abstract led to the exclusion of 3715 records. Full text was sought for 66 records, of which seven could not be retrieved. From the 59 eligible studies, a total of 20 studies were excluded following full-text review. Reasons for exclusion included: the absence of reported prevalence (n = 2) [25,26]; data collected before 2011 (n = 2) [27,28]; the study’s participants being restricted to a specific age group and not being representative of a whole population (n = 1) [29]; the population were children (n = 3) [30,31,32]; population was mixed and data could not be separated for the included population i.e., including Saudis and non-Saudis (n = 3) [33,34,35] or adults and children (n = 1) [36]; and studies with a scope irrelevant to the objectives of this review (n = 4) [37,38,39,40] or presenting findings that have been reported more than once (n = 4) [34,38,41,42]. Finally, 39 studies were included in this review (Figure 1 and Table 1).

### 3.1. Age, Gender, and Temporal Influences on the Prevalence of Obesity and Overweight among Young Adults

**Figure 2:**
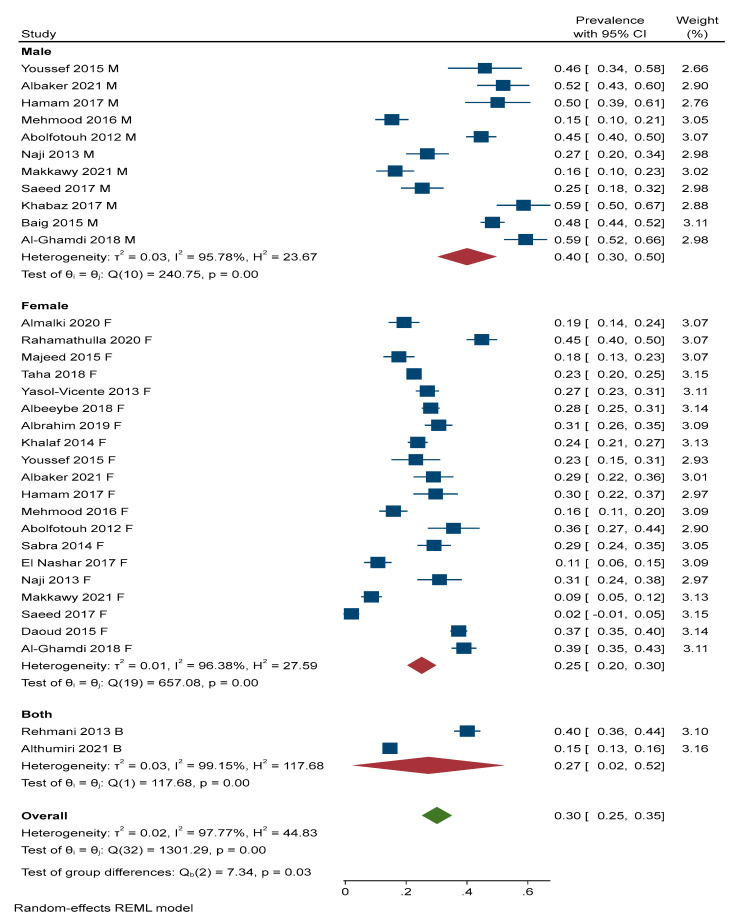
Prevalence of obesity among young adults by gender. M = Male, F = Female, B = Both (male and female) [41,42,43,44,45,46,47,48,49,50,51,52,53,54,55,56,57,58,59,60,61,62,63].

**Figure 3:**
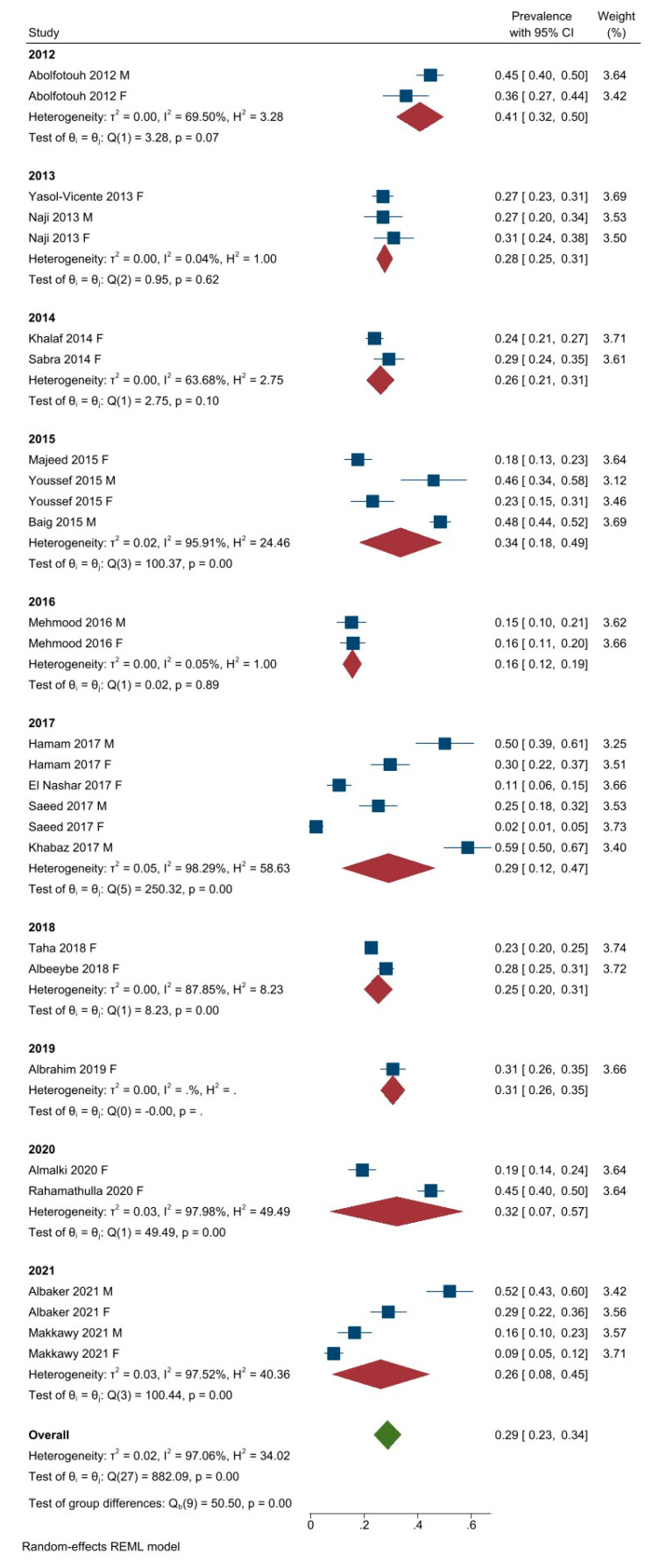
Prevalence of obesity among young adults by year. M = Male, F = Female, B = Both (male and female) [41,42,43,44,45,46,47,48,49,50,51,52,53,54,55,56,57,58,59,60,61,62,63,64].

The pooled prevalence of obesity and overweight in the age group of ≤25 years old, including both genders, was 30% (Figure 2 and Table 1). However, it was higher among males (40%) compared to young females (25%). Furthermore, there is obvious temporal variation in the prevalence of obesity and overweight among young adults, which dropped from 41% in 2012 to 24% in 2021 (Figure 3).

### 3.2. Age, Gender, and Temporal Influences on the Prevalence of Obesity and Overweight among Adults, Mid-Life, and Old People

**Figure 4:**
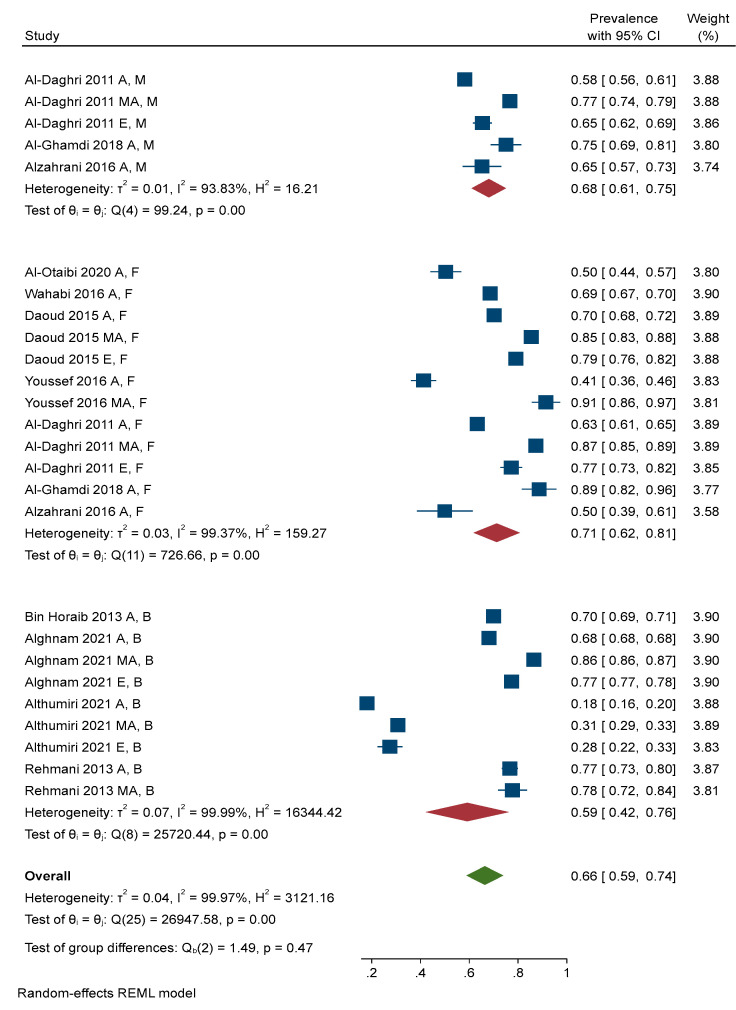
Prevalence of obesity for all groups, excluding young adults, by gender. A M = Adult males, AF = Adult females, AB = Adult males and females, MA M = middle-aged males, MA F = Middle-aged females, MA B = Middle-aged males and females, EM = Old males, EF = Old females, EB = Old males and females [52,62,63,64,65,66,67,68,69,70,71].

**Figure 5:**
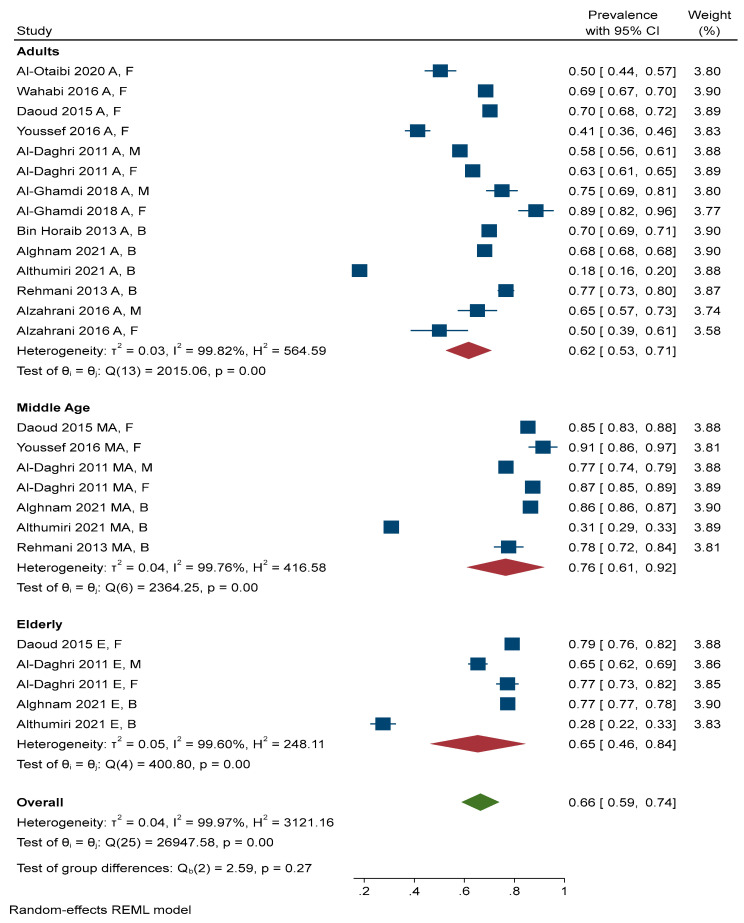
Prevalence of obesity for all participants excluding young adults by age group. A M = Adult males, AF = Adult females, AB = Adult males and females, MA M = middle-aged males, MA F = Middle-aged females, MA B = Middle-aged males and females, EM = Old males, EF = Old females, EB = Old males and females [52,62,63,64,65,66,67,68,69,70,71].

**Figure 6:**
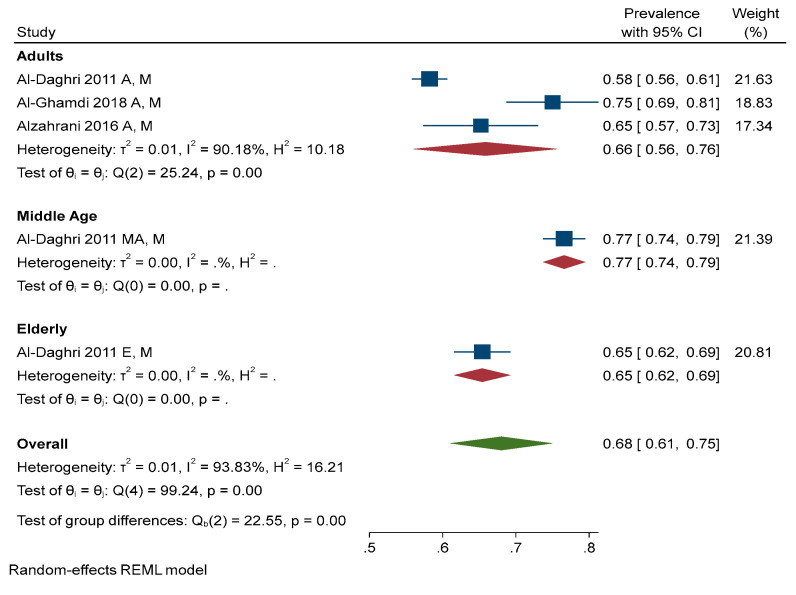
Prevalence of obesity for all participants excluding young adults by age group among males. AM = Adult males, MA M = Middle-Aged males, EM = Old males [52,65,66].

**Figure 7:**
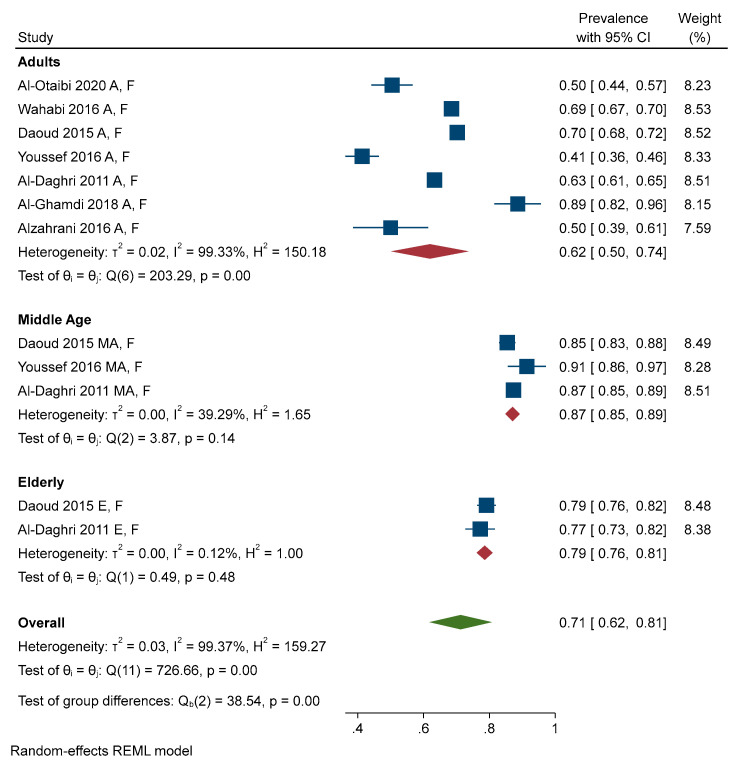
Prevalence of obesity for all participants excluding young adults by age group among females. AF = Adult females, MA F = Middle-aged females, EF = Old females [52,62,65,66,67,68,69].

**Figure 8:**
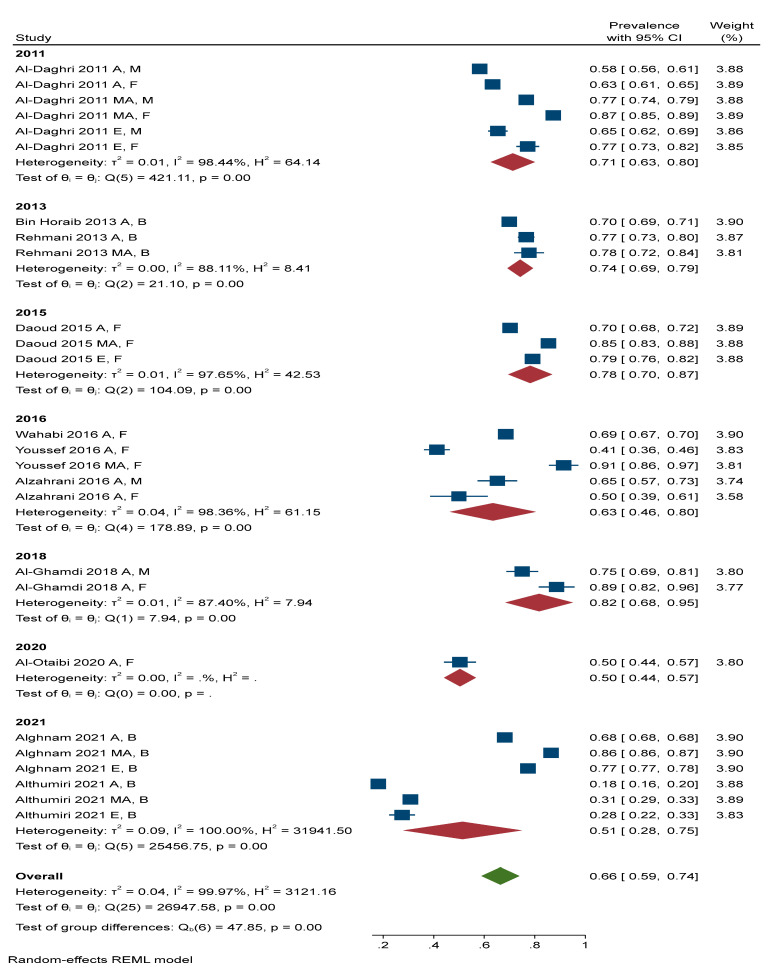
Prevalence of obesity for all participants excluding young adults by year of publication. A M = Adult males, AF = Adult females, AB = Adult males and females, MA M = middle-aged males, MA F = Middle-aged females, MA B = Middle-aged males and females, EM = Old males, EF = Old females, EB = Old males and females [52,62,63,64,65,66,67,68,69,70,71].

The overall pooled prevalence rate of obesity and overweight in the age groups older than 25 years (adults, mid-life, and old people) including both genders was 66%, with nearly similar prevalence among males (68%) and females (71%) (Figure 4 and Table 1). Considering specific age groups, similar rates were observed among both adults and old people (62% and 65%, respectively). However, the pooled prevalence was highest among adults in the mid-life group (76%), showing an increase of nearly 10% from other age categories (Figure 5). Further analysis of the prevalence in this group by gender (Figure 6 and Figure 7 and Table 1) showed that mid-life women had the highest prevalence among all groups (87%), compared to 77% among males in the same age group. The same difference in prevalence between both genders persisted in old females compared to old males (79% vs. 65%, respectively) (Figure 6 and Figure 7). There is a noticeable drop in the pooled prevalence of overweight and obesity among adults aged over 25 years of 28% in the year 2021 compared to that reported in the year 2011 (Figure 8 and Table 1).

### 3.3. Prevalence of Obesity and Overweight by Geographic Area

**Figure 9:**
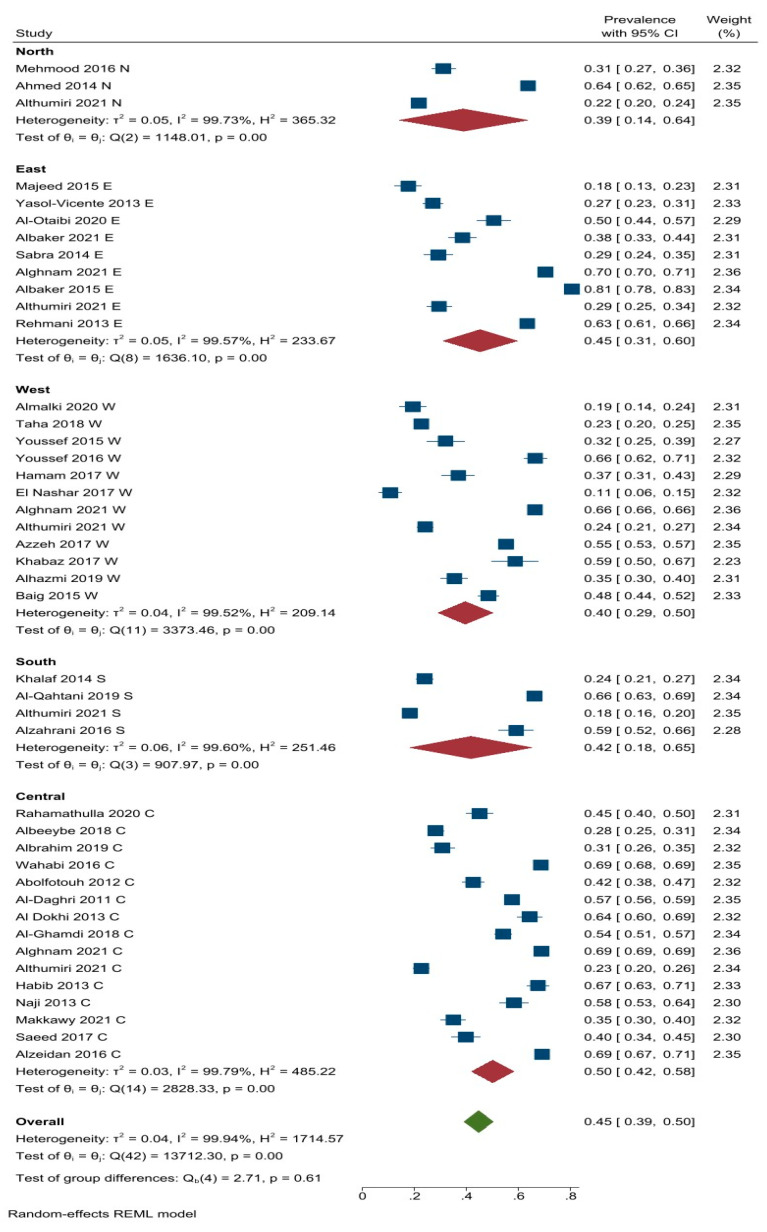
Prevalence of obesity and overweight by geographic area. N = North, E = East, W = West, S = South, C = Central [41,42,43,44,45,46,47,48,49,50,51,52,53,54,55,56,57,58,59,60,61,63,64,65,66,67,68,69,71,72,73,74,75,76,77,78,79].

**Figure 10:**
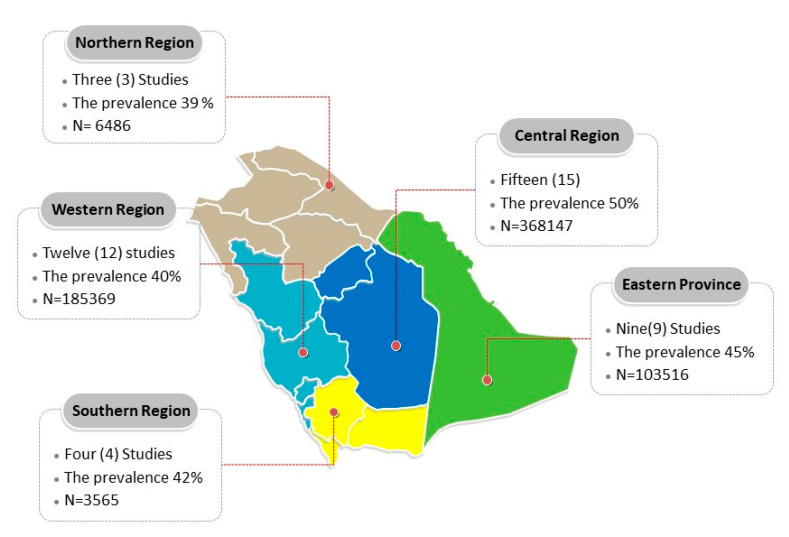
Prevalence of obesity and overweight across the five geographic areas.

Two studies were excluded from this analysis because the prevalence was reported at the national level and data could not be separated [62,70]. The group differences among the five geographical regions were non-significant (X^2^ = 2.71, *p*-value = 0.607). The central region had the highest prevalence (50%), followed by the eastern region (45%), southern region (42%), western region (40%), and northern region (39%) (Figure 9 and Figure 10 and Table 1).

**Table 1:** Summary of the Included Studies.

No	Study ID	Design	Setting	Region/City	Population	Sample Size	Age (Years) Range (Mean ± SD)	Prevalence
1	Almalki 2020 [53]	Cross-sectional	University	Western/Jeddah	Female university students	Total = 232	18–27 (20.98 ± 1.48)	Overall (19.3%)
2	Abolfotouh 2012 [46]	Cross-sectional	University	Central/Riyadh	University students	Total = 501 F = 118 (23.5%)	18–26	Overall (42.5%) M (44.7%) F (35.6%)
3	Ahmed 2014 [72]	Cross-sectional	Primary health care centers	Northern/Hail	General population	Total = 5000	<25–71+ (43.5 ± 18.7) M (44.6 ± 20.2) F (42.3 ± 16.9)	Overall (63.6%) M (56.2%) F (71%)
4	Al Dokhi 2013 [73]	Cross-sectional	Population-based	Central	Healthy adults	Total = 411 F = 111 (27%)	18–72 (36.91 ± 15.22)	Overall (64.2%) M (57.4%) F (71%)
5	Albaker 2015 [74]	Cross-sectional	University Hospital	Eastern	General population	Total = 711 F = 356 (50.1%)	18–60 (40 ± 12.9)	Overall (80.5%)
6	Albaker 2021 [43]	Cross-sectional	University	Eastern/Dammam	University students	Total = 310 F = 183 (59%)	18.45 ± 0.8	Overall (38.4%) M (51.9%) F (29%)
7	Albeeybe 2018 [57]	Cross-sectional	University	Central/Riyadh	Female university students	Total = 907	(20.9 ± 1.8)	Overall (28.1%)
8	Albrahim 2019 [58]	Cross-sectional	University	Central/Riyadh	Female university students	Total = 396	18–24 (20.1 ± 1.55)	Overall (30.7%)
9	Al-Daghri 2011 [65]	Cohort study	Primary healthcare centers	Central/Riyadh	General population	Total = 6630 F = 3640 (54.9%)	18–80	Overall (57.4%) By age and gender: 18–45 [M (58.2%), F (63.3%)] 46–60 [M (67.6%), F (78.3%)] 61–80 [M (65.4%), F (77.2%)]
10	Al-Ghamdi 2018 [52]	Cross-sectional	Population-based	Central/Al Kharj	General population	Total = 1019 F = 638 (63%)	18–67	Overall (54.3%) By age and gender: 18–29: [M (59%), F (39%)] 30–67: [M (75%), F (89%)]
11	Alghnam 2021 [71]	Cross-sectional	Population-based	Central, western, and eastern	Patients	Total = 573,698	17 years and older	Overall (68.6%) By age: 17–25 (38.5%) 26–45 (68%) 46–64 (86.4%) ≥65 (77.6%)
12	Alhazmi 2019 [75]	Cross-sectional	University	Western/AL Madinah Al Monawarah	University students	Total = 342 F = 171 (50%)	≤22 years (64.6%) > 22 years (35.4%)	Overall (35.3%)
13	Al-Otaibi 2020 [67]	Cross-sectional	Hospital	Eastern/Al-Ahsa	Pregnant women	Total = 238	20–40 (29.2 ± 5.1)	Overall (50.4%)
14	Al-Qahtani 2019 [76]	Cross-sectional	Primary health care centers	Southwestern	Healthy adults	Total = 1238 F = 410 (34%)	Not reported	Overall (66%)
15	Althumiri 2021 [64]	Cross-sectional	Population-based	National 13 Regions	Healthy adults	Total = 4709 F = 2358 (50.1%)	18–90 (36.4 ± 13.5)	Overall (24.7%) By age: 18–19 (14.1%) 20–29 (14.8%) 30–39 (18.1%) 40–49 (29.8%) 50–59 (32.8%) 60–(27.6%)
16	Alzahrani 2016 [66]	Cross-sectional	Hospitals and family medicine centers	Southern/Aseer	Resident physicians	Total = 211 F = 33.2%	≤25–>30 (27.9 ± 2.6)	Overall (59%) M (65.2%) F (50%)
17	Alzeidan 2016 [77]	Cross-sectional	University	Central/Riyadh	University employees and families	Total = 3063 F = 1907 (62.3%)	18–≥60 (38.58 ± 14.09)	Overall (69%)
18	Azzeh 2017 [78]	Cross-sectional	Population-based	Western/Mecca, Jeddah, and Altaif	Healthy adults	Total = 2548 F = 1125 (44.2%)	Overall 18–60 (29.1 ± 8.5) F (28.3 ± 9.1) M (29.7 ± 8)	Overall (55.1%) M (62.8%) F (45.3%)
19	Baig 2015 [51]	Cross-sectional	University	Western/Jeddah	Male University Students	Total = 610	22.40 ± 3.90	Overall (48.4%)
20	Bin Horaib 2013 [70]	Cross-sectional	Population based	Nationwide five regions of KSA (Eastern, northern, southern, western, and central)	Healthy adult (Military personnel)	Total = 10500	<30–50 (34.12 ± 7.25)	Overall (62.4%)
21	Daoud 2015 [62]	Cross-sectional	National Households and primary healthcare clinics	All regions (13 health regions)	Adult women	Total = 5482	15–≥65	Overall (61.5%) By age: 15 –24 (37.3%) 25–44 (70%) 45–60 (85%) >60 (79%)
22	El Nashar 2017 [61]	Cross-sectional	University	Western/AL Madinah Al Monawarah	Female students	Total = 186	Preparatory university students	Overall (10.7%)
23	Habib 2013 [79]	Cross-sectional	Population-based	Central	Healthy adults	Total = 530 F = 167 (31.5%)	18–72 (36.9 ± 15.2) F = 36.7 ± 9.5 M = 38.8 ± 15.6	Overall (67.4%)
24	Hamam 2017 [44]	Cross-sectional	University	Western/Taif	University students	Total = 228 F = 148 (64.9%)	1st–6th years of university	Overall (36.8%) M (50.1%) F (29.7%)
25	Khabaz 2017 [50]	Cross-sectional	University,	Western/Jeddah	University Students	Total = 116	18–26 years	Overall (58.6%)
26	Khalaf 2014 [59]	Cross-sectional	University	Southwestern	Female university students	Total = 663	18–25 (20.4 ± 1.5)	Overall (23.8%)
27	Majeed 2015 [41]	Cross-sectional	University	Eastern/Dammam	Female university students	Total = 215	(19.27 ± 0.95)	Overall (17.6%)
28	Makkawy 2021 [48]	Cross-sectional	University	Central/Riyadh	University Students	Total = 401 F = 267 (66.6%)	(22.2 ± 2.9)	Overall (34.9%) M (16.4%) F (8.6%)
29	Mehmood 2016 [45]	Cross-sectional	University	Northern/Ar’ar	University students	Total = 405 F = 236 (58.3%)	19–25 (21.49 ±1.59)	Overall (31.1%) M (15.3%) F (15.8%)
30	Naji 2013 [47]	Cross-sectional	University	Central/Al-Majmaah	University students	Total = 303 F = 151 (49.8%)	Not reported	Overall (58.1%) M (27%) F (31%)
31	Rahamathulla 2020 [54]	Cross-sectional	University	Central/Wadi Al Dawaser	Female university students	Total = 374	(20.6 ± 1.26)	Overall (44.9%)
32	Rehmani 2013 [63]	Cross-sectional	Population-based	Eastern	Healthy adolescents and adults	Total = 1339 F = 570 (42.6%)	≥15 (30.7 ± 12.7)	Overall (63.3%) M (67.9%) F (57%) By age categories: 15–24 (40.1%) 25–34 (78.3%) 35–44 (76.7%) 45–54 (80.1%) ≥55 (60.9%)
33	Sabra 2014 [60]	Cross-sectional	University	Eastern/Dammam	Female university students	Total = 260	18–≥22	Overall = (29.2%)
34	Saeed 2017 [49]	Cross-sectional	University	Central/Riyadh	University students	Total = 281	21.26 ± 1.21	Overall (39.7%) M (25.3%) F (2.1%)
35	Taha 2018 [55]	Cross-sectional	University	Western/Taif	Female university students	Total = 1200	17–33 Median = 21	Overall (22.6%)
36	Wahabi 2016 [68]	Cohort study	Hospitals	Central/Riyadh	Pregnant women and maternity population	Total = 14568	(29 ± 5.9)	Overall (68.5%)
37	Yasol-Vicente 2013 [56]	Cross-sectional	University	Eastern/Jubail	Female university students	Total = 514	Preparatory + Bachelor students	Overall (27%)
38	Youssef 2015 [42]	Cross-sectional	University	Western/AL-Madina Al-Monawarah	University students	Total = 165 F = 104 (63%)	(20.5 ± 0.1)	Overall (31.9%) M (45.9%) F (23.2%)
39	Youssef 2016 [69]	Cross-sectional	Population-based	Western/AL-Madina Al-Monawarah	Adult women	Total = 448	18–60	By age categories: 18–39 (41.3%) 40–60 (91.4%)

M: Male, F: Female.

## 4. Discussion

The current study investigated the influences of age, gender, temporality, and geographical area on the prevalence of obesity and overweight among Saudi Arabian adults. The findings revealed that the pooled prevalence among adults aged over 25 years was more than double that of young adults (66% vs. 30%). While the pooled prevalence of obesity and overweight was higher in males compared to females in the young adults’ group, it was higher in females than in males in the mid-life and old age groups. In addition, the mid-life group was associated with the highest prevalence rates for both genders, but there was a higher prevalence among females than males in this age group. However, there was a noticeable drop in the prevalence of obesity and overweight among all age groups between the year 2011 and 2021. This study did not show a difference in the prevalence of overweight/obesity between the five geographical areas of the Kingdom.

The pooled prevalence of obesity among young adults in this study was 30%, which is in line with the findings of other studies in both developed and developing countries [80]. Similar trends in the prevalence of overweight and obesity by gender and age have been reported in other countries of the Eastern Mediterranean Region [3,81]. Estimates of the prevalence of obesity and overweight in USA and UK were 22% to 35%, respectively, among the 18–23 years old age group [82,83]. Peltzer et al. investigated the rate of obesity among university students from 22 low- and middle-income countries and reported a similar overall rate of obesity of 22% [84]. In agreement with several studies, mainly from Asia [85,86,87], young adult males showed higher prevalence rates of overweight/obesity compared to females. However, this finding is not consistent all over the world, as reported by other investigators [84].

Several factors may contribute to such gender differences in this age group. Taking into consideration that the majority of young adults are university students, as well as the conservative culture in Saudi Arabia, males are more likely than females to live away from their families to pursue their undergraduate study in different universities, which renders them more likely to gain weight by depending mainly on fast food and soft drinks for their diet [88,89].

Another explanation comes from gender discrepancy of body image perception. Several studies have found that males underestimate their weights compared to females, and that overweight/obese males perceive being overweight to be the ideal body image, whereas females perceive a normal weight to be the ideal body image [90,91]. This may lead young men to gain weight in order to reach their ‘ideal’ buff image, while young women will desire to lose weight and maintain a thin body image [80].

Similar to other reports in the literature, this study showed an increase in the prevalence of overweight/obesity with age [92]. Changes in lifestyle which have already started during young adulthood, such as increased consumption of fast food, skipping breakfast, and snacking, all increase the risk of being overweight/obese later during adult and mid-life [93,94,95]. In addition, the shift from active to sedentary lifestyles inside and outside the workplace, the lack of time to practice physical activity due to work or social and family responsibilities, together with decreased metabolism with age all contribute to less activity and more obesity.

Females in adult and mid-life age experience more physiological changes due to pregnancy and childbearing, which, along with the above-mentioned factors, makes women in their reproductive age more obese than men in the same age groups. Alotibi et al. examined the pre-pregnancy weight of nulliparous women and found that the prevalence of obesity in this group of women was only 14.7% compared to 64% in women who had two or more children [67]. Wahabi et al. 2019 investigated postpartum weight retention in Saudi Arabian women and showed that 35% of the investigated population had ≥7 kg of extra weight retained one year following delivery [96].

Moreover, although more than 40% of Saudi Arabian women are university graduates, they have higher unemployment rates at 80–86% [67,68], which contributes to physical inactivity following completion of their education [77]. These factors can explain the dominance of obesity in females during the adult, mid-life, and old age stages compared to males in the current study.

We were rather surprised by the decreased prevalence of overweight/obesity we observed in this study between the years 2011 and 2021. Studies that link the reduction of the prevalence of obesity and overweight in Saudi Arabia to certain interventions on a national scale, are scarce [64]. Nevertheless, many new regulations that promote a healthier lifestyle have been introduced to Saudi Arabia on a national level, which may have contributed to the current reduction in obesity/overweight found in this study. Example of these changes include new regulations that allowed female fitness centers to open in the country, and physical activity classes in female schools which were introduced in 2017 [97,98]. Furthermore, as part of the Vision 2030 plan, the government initiated quality of life programs and resources to encourage community participation in exercise as well as healthy lifestyles [99]. The introduction of legislation requiring printing a meal’s calories on restaurant menus at the beginning of 2019, with the intention to increase awareness of the importance of the amount of calories each type of food contains in the Saudi Arabian community, is another pertinent intervention [100]. Excise taxes of 50% and 100% were introduced on sugar-sweetened and carbonated drinks and energy drinks, respectively, in early 2019 [101].

The increase in the treatment of obesity, including bariatric surgery, may have increased the awareness of the community at large about obesity as a chronic disease and a risk factor for other chronic diseases, especially with coverage by press and social media [102,103].

In this study there was no significant difference in the pooled prevalence of obesity/overweight between the regions of Saudi Arabia, and an overall prevalence of 45%. This may be due to the balanced distribution of health care and social facilities in the Kingdom. However, most of the studies in this review were conducted in urban cities, rather than in rural areas, which share the same standard of living, lifestyle and availability of restaurants that provide fast food and high-calorie drinks. In addition, the new large geographical regions we used in this study may have obscured the small variations in prevalence of obesity/overweight that were noticed in other studies using smaller geographical regions [10].

One of the many programs and interventions the Kingdom has initiated to address the problem of obesity among the different sectors of the community was the approval of national clinical practice guidelines for the management of overweight and obesity, which were published in 2016 [64,104]. However, the ACTION International Observation Study (ACTION-IO), which included healthcare professionals and individuals with obesity from many countries around the world, including Saudi Arabia, found several barriers to effective obesity healthcare, which needs a set of tailored interventions, such as education and training [18,105]. Many of the barriers identified in the ACTION-IO study were related to a lack of communication and trust between healthcare providers and people living with obesity, which delayed timely effective interventions [18]. In addition, the study documented many misconceptions among both groups, including that people with obesity believe they should accept complete responsibility for their own weight loss, and that fewer than 50% of healthcare providers believed genetics were barriers to obesity management [18].

### 4.1. Implications for Research (Research Gaps) and Practice

In line with the national promotion of public health and preventive medicine in Saudi Arabia, more studies are required to further explore the epidemiology and determinants of overweight and obesity and propose strategies to address the challenges and barriers for obesity prevention and management [106].

Future epidemiological studies should stratify epidemiological findings based on gender and age to tailor effective intervention for each group of the population based on their characteristics and specific platforms for communication. Such stratification will help in the detection of the group mostly in need of interventions (e.g., women at reproductive age, postpartum women).

Further studies are needed to explore the viewpoints of the Saudi Arabian community at large and the individuals living with obesity regarding the social, economic, and medical aspects of overweight/obesity. Such information will facilitate designing and implementing effective educational programs pertaining to obesity prevention and management with partnership between the community and healthcare providers [104,107].

Obesity varies with socioeconomic, ethnic, and other factors, and the findings of this review indicate the need for studies to assess whether obesity prevalence has a different distribution at a smaller geographic level than the regional level, for instance, between urban and rural areas. Such knowledge will advise better allocation of resources and targeted interventions [108].

Social media and the press should be employed to increase the awareness of the community about obesity as a chronic disease and as a risk factor for other disease, and channels for seeking medical attention and advice should be established and made available and easily accessible [109].

All the implemented and the proposed programs for obesity in Saudi Arabia should include surveillance systems and evaluation plans to monitor the progress of effective interventions and facilitate audits for maximum cost-effectiveness [104].

Similar to other medical conditions and chronic diseases, individuals with obesity should be the center of care [110].

Undergraduate and graduate medical curricula should include updated and evidence-based information on the epidemiology, etiology, risk factors, management, and prevention of obesity at the individual and national levels [111].

### 4.2. Strength and Limitation of this Study

This review is the first report to provide data on the prevalence of obesity by age and gender and geographical region in Saudi Arabia. It provides valuable information on specific sectors of the populations to be targeted with tailored effective interventions [20,112]. Despite implementing several promising policies in Saudi Arabia, it is not yet clear how and to what extent they have impacted each group of the population [113]. This scoping review is the first step in planning age-and-gender-specific strategies.

However, the results of this review should be approached with caution due to the high heterogeneity associated with pooling the data. This may be due to differences in the study designs, but performance bias cannot be excluded, especially considering that we did not conduct critical appraisal for any of the included studies.

## 5. Conclusions

Despite the noticeable drop in the prevalence of obesity in the Saudi community the prevalence of high BMI is high in Saudi Arabia irrespective of age, gender, or geographical location. Mid-life women have the highest prevalence of high BMI, which should make them the focus of a tailored strategy for intervention. Further research is needed to investigate which are the most effective interventions to address obesity in the country.

## Data Availability

All relevant data are within the manuscript and its supporting information files.

## Supplementary Materials

The following supporting information can be downloaded at: https://www.mdpi.com/article/10.3390/healthcare11081143/s1, Table S1: Search strategies title.
