# Supplementary material for: The Impact of Age, Gender, Temporality, and Geographical Region on the Prevalence of Obesity and Overweight in Saudi Arabia: Scope of Evidence
Source: Healthcare (Basel). 2023 Apr 15;11(8):1143. doi: 10.3390/healthcare11081143 (PMC10137821; doi:10.3390/healthcare11081143)
Supplement: Supplementary file 1 [file healthcare-11-01143-s001.zip › healthcare-2309823-supplementary.pdf]

Search strategies:

**CINAHL**		
#	**Query**	**Results**
S7	S3 AND S6	599
S6	S4 OR S5	327,312
S5	TI ((Obes* OR overweight* OR (over W0 (nourish* OR nutrit* OR weight*)) OR (body W0 mass W0 index*) OR (weight W0 (reduc* OR lo#s* OR decreas* OR watch* OR control*)) OR overnutrition* OR over-nutrition* OR overnourish* OR over-nourish OR adipos* OR (waist W0 hip W0 ratio#) OR (skin W0 fold W0 thickness) OR overeat* OR (over W0 eat*) OR ((wast* OR (night W0 eating)) W0 (syndrome* OR disorder*)) OR (growth W0 falter*) OR ((eating OR feeding OR appetite OR nutrition*) W0 disorder#) OR (weight W0 length W0 percentile#) OR Hyperphagia* OR polyphagia* OR BMI OR (sagittal W0 abdominal W0 diameter#) OR (abdominal W0 (fat# OR (diameter W0 index) OR height)) OR (waist W0 (circumference# OR (to W0 (height OR hip)) OR height OR hip)) OR (lipid W0 accumalat* W0 product#) OR hypernutrition* OR hyper-nutrition*)) OR AB ((Obes* OR overweight* OR (over W0 (nourish* OR nutrit* OR weight*)) OR (body W0 mass W0 index*) OR (weight W0 (reduc* OR lo#s* OR decreas* OR watch* OR control*)) OR overnutrition* OR over-nutrition* OR overnourish* OR over-nourish OR adipos* OR (waist W0 hip W0 ratio#) OR (skin W0 fold W0 thickness) OR overeat* OR (over W0 eat*) OR ((wast* OR (night W0 eating)) W0 (syndrome* OR disorder*)) OR (growth W0 falter*) OR ((eating OR feeding OR appetite OR nutrition*) W0 disorder#) OR (weight W0 length W0 percentile#) OR Hyperphagia* OR polyphagia* OR BMI OR (sagittal W0 abdominal W0 diameter#) OR (abdominal W0 (fat# OR (diameter W0 index) OR height)) OR (waist W0 (circumference# OR (to W0 (height OR hip)) OR height OR hip)) OR (lipid W0 accumalat* W0 product#) OR hypernutrition* OR hyper-nutrition*)) OR MW ((Obes* OR overweight* OR (over W0 (nourish* OR nutrit* OR weight*)) OR (body W0 mass W0 index*) OR (weight W0 (reduc* OR lo#s* OR decreas* OR watch* OR control*)) OR overnutrition* OR over-nutrition* OR overnourish* OR over-nourish OR adipos* OR (waist W0 hip W0 ratio#) OR (skin W0 fold W0 thickness) OR overeat* OR (over W0 eat*) OR ((wast* OR (night W0 eating)) W0 (syndrome* OR disorder*)) OR (growth W0 falter*) OR ((eating OR feeding OR appetite OR nutrition*) W0 disorder#) OR (weight W0 length W0 percentile#) OR Hyperphagia* OR polyphagia* OR BMI OR (sagittal W0 abdominal W0 diameter#) OR (abdominal W0 (fat# OR (diameter W0 index) OR height)) OR (waist W0 (circumference# OR (to W0 (height OR hip)) OR height OR hip)) OR (lipid W0 accumalat* W0 product#) OR hypernutrition* OR hyper-nutrition*))	306,244
S4	(MH "Obesity") OR (MH "Pediatric Obesity") OR (MH "Body Weight Changes") OR (MH "Weight Gain") OR (MH "Wasting Syndrome+") OR (MH "Overnutrition") OR (MH "Obesity, Maternal") OR (MH "Obesity, Morbid") OR (MH "Pickwickian Syndrome") OR (MH "Hyperphagia") OR (MH "Eating Disorders") OR (MH "Body Weight") OR (MH "Body Weight Changes") OR (MH "Adipose Tissue Distribution") OR (MH "Body Mass Index") OR (MH "Waist Circumference") OR (MH "Waist-Hip Ratio") OR (MH "Skinfold Thickness") OR (MH "Weight ...	206,440
S3	S1 OR S2	7,885
S2	TI ((saudi* OR KSA OR riyadh OR Riyad OR Jedda* OR Jedde* OR “El Khobar” OR “Al Khobar” OR El-Khobar OR Al-Khobar OR Khobar OR Hijaz OR Dammam OR Medina* OR Mekka* OR Makka* OR Abha OR KAUST)) OR AB ((saudi* OR KSA OR riyadh OR Riyad OR Jedda* OR Jedde* OR “El Khobar” OR “Al Khobar” OR El-Khobar OR Al-Khobar OR Khobar OR Hijaz OR Dammam OR Medina* OR Mekka* OR Makka* OR Abha OR KAUST)) OR MW ((saudi* OR KSA OR riyadh OR Riyad OR Jedda* OR Jedde* OR “El Khobar” OR “Al Khobar” OR El-Khobar OR Al-Khobar OR Khobar OR Hijaz OR Dammam OR Medina* OR Mekka* OR Makka* OR Abha OR KAUST))	7,885
S1	(MH "Saudi Arabia")	5,910
Cochrane		
ID	Search Hits	**Results**
#1	MeSH descriptor: [Saudi Arabia] this term only	198
#2	((saudi* OR KSA OR riyadh OR Riyad OR Jedda* OR Jedde* OR "El Khobar" OR "Al Khobar" OR El-Khobar OR Al-Khobar OR Khobar OR Hijaz OR Dammam OR Medina* OR Mekka* OR Makka* OR Abha OR KAUST)):ti,ab,kw	1259
#3	#1 OR #2	1259
#4	MeSH descriptor: [Obesity] this term only	12580
	#5 MeSH descriptor: [Pediatric Obesity] this term only 1438	

#6	MeSH descriptor: [Hyperphagia] explode all trees	738
#7	MeSH descriptor: [Weight Gain] this term only	2678
#8	MeSH descriptor: [Body Weight] this term only	8780
#9	MeSH descriptor: [Overweight] this term only	5667
#10	MeSH descriptor: [Body Fat Distribution] explode all trees	1022
#11	MeSH descriptor: [Body Mass Index] this term only	10782
#12	MeSH descriptor: [Sagittal Abdominal Diameter] this term only	0
#13	MeSH descriptor: [Waist Circumference] explode all trees	1128
#14	MeSH descriptor: [Waist-Height Ratio] this term only	10
#15	MeSH descriptor: [Skinfold Thickness] this term only	324
#16	MeSH descriptor: [Waist-Hip Ratio] this term only	274
#17	MeSH descriptor: [Weight Reduction Programs] this term only	856
#18	MeSH descriptor: [Body Fat Distribution] explode all trees	1022
#19	MeSH descriptor: [Nutrition Disorders] this term only	498
#20	MeSH descriptor: [Child Nutrition Disorders] this term only	256
#21	MeSH descriptor: [Infant Nutrition Disorders] this term only	143
#22	MeSH descriptor: [Feeding and Eating Disorders] this term only	854
#23	MeSH descriptor: [Night Eating Syndrome] this term only	0
#24	((Obes* OR overweight* OR (over NEXT/1 (nourish* OR nutrit* OR weight*)) OR (body NEXT/1 mass NEXT/1 index*) OR (weight NEXT/1 (reduc* OR lo?s* OR decreas* OR watch* OR control*)) OR overnutrition* OR over-nutrition* OR overnourish* OR over-nourish OR adipos* OR (waist NEXT/1 hip NEXT/1 ratio?) OR (skin NEXT/1 fold NEXT/1 thickness) OR overeat* OR (over NEXT/1 eat*) OR ((wast* OR (night NEXT/1 eating)) NEXT/1 (syndrome* OR disorder*)) OR (growth NEXT/1 falter*) OR ((eating OR feeding OR appetite OR nutrition*) NEXT/1 disorder?) OR (weight NEXT/1 length NEXT/1 percentile?) OR Hyperphagia* OR polyphagia* OR BMI OR (sagittal NEXT/1 abdominal NEXT/1 diameter?) OR (abdominal NEXT/1 (fat? OR (diameter NEXT/1 index) OR height)) OR (waist NEXT/1 (circumference? OR (to NEXT/1 (height OR hip)) OR height OR hip)) OR (lipid NEXT/1 accumalat* NEXT/1 product?) OR hypernutrition* OR hyper-nutrition*)):ti,ab,kw	115768
#25	{OR #4-#23}	32980
#26	#3 AND #25	22
Embase		
#13	#1 AND #12	3820
#12	#2 OR #3 OR #4 OR #5 OR #6 OR #7 OR #8 OR #9 OR #10 OR #11	1841413
#11	((waist NEXT/1 (circumference$ OR 'to height' OR 'to hip' OR height OR hip)):ti,ab,kw) OR ((lipid NEXT/1 accumalat* NEXT/1 product$):ti,ab,kw) OR hypernutrition*:ti,ab,kw OR 'hyper nutrition*':ti,ab,kw	62323
#10	((sagittal NEXT/1 abdominal NEXT/1 diameter$):ti,ab,kw) OR ((abdominal NEXT/1 (fat$ OR 'diameter index' OR height)):ti,ab,kw)	9755
#9	((weight NEXT/1 length NEXT/1 percentile$):ti,ab,kw) OR hyperphagia*:ti,ab,kw OR polyphagia*:ti,ab,kw OR bmi:ti,ab,kw	364721
#8	((growth NEXT/1 falter*):ti,ab,kw) OR (((eating OR feeding OR appetite OR nutrition*) NEXT/1 disorder$):ti,ab,kw)	34379
#7	((wast* OR 'night eating') NEXT/1 (syndrome* OR disorder*)):ti,ab,kw	3340
#6	'skin fold thickness':ti,ab,kw OR overeat*:ti,ab,kw OR ((over NEXT/1 eat*):ti,ab,kw)	5914
#5	overnutrition*:ti,ab,kw OR 'over nutrition*':ti,ab,kw OR overnourish*:ti,ab,kw OR 'over nourish':ti,ab,kw OR adipos*:ti,ab,kw OR ((waist NEXT/1 hip NEXT/1 ratio$):ti,ab,kw)	173997
#4	((body NEXT/1 mass NEXT/1 index*):ti,ab,kw) OR ((weight NEXT/1 (reduc* OR lo$s* OR decreas* OR watch* OR control*)):ti,ab,kw)	470462
#3	obes*:ti,ab,kw OR overweight*:ti,ab,kw OR ((over NEXT/1 (nourish* OR nutrit* OR weight*)):ti,ab,kw)	545579
#2	obesity'/exp OR 'hyperphagia'/de OR 'body weight gain'/exp OR 'body weight'/exp OR 'body mass'/de OR 'sagittal abdominal diameter'/de OR 'waist circumference'/de OR 'waist to height ratio'/de OR 'skinfold thickness'/exp OR 'waist hip ratio'/de OR 'weight loss program'/de OR	1567779

	'body fat distribution'/de OR 'nutritional disorder'/de OR 'eating disorder'/de OR 'binge eating disorder'/exp OR 'obesity hypoventilation syndrome'/de	
#1	'saudi'/de OR 'saudi arabia'/de OR saudi*:ti,ab,kw OR ksa:ti,ab,kw OR riyadh:ti,ab,kw OR riyad:ti,ab,kw OR jedda*:ti,ab,kw OR jedde*:ti,ab,kw OR 'el-khoba':ti,ab,kw OR 'al-khobar':ti,ab,kw OR 'el khobar':ti,ab,kw OR 'al khobar':ti,ab,kw OR khobar:ti,ab,kw OR hijaz:ti,ab,kw OR dammam:ti,ab,kw OR medina*:ti,ab,kw OR mekka*:ti,ab,kw OR makka*:ti,ab,kw OR abha:ti,ab,kw OR kaust:ti,ab,kw	37447
Ovid MEDLINE(R) and Epub Ahead of Print, In-Process, In-Data-Review & Other Non-Indexed Citations and Daily <1946 to January 31, 2022>		
1	Saudi Arabia/	(15550)
2	(saudi* or KSA or riyadh or Riyad or Jedda* or Jedde* or "El Khobar" or "Al Khobar" or El-Khobar or Al-Khobar or Khobar or Hijaz or Dammam or Medina* or Mekka* or Makka* or Abha or KAUST).mp.	(30054)
3	1 or 2	(30054)
4	Obesity/ or Pediatric Obesity/ or exp Hyperphagia/ or Weight Gain/ or Body Weight/ or Overweight/ or exp body fat distribution/ or body mass index/ or sagittal abdominal diameter/ or exp waist circumference/ or waist-height ratio/ or skinfold thickness/ or waist-hip ratio/ or Weight Reduction Programs/ or exp body fat distribution/ or nutrition disorders/ or child nutrition disorders/ or infant nutrition disorders/ or "feeding and eating disorders"/ or night eating syndrome/	(539272)
5	(Obes* or overweight* or (over adj (nourish* or nutrit* or weight*)) or (body adj mass adj index*) or (weight adj (reduc* or lo?s* or decreas* or watch* or control*)) or overnutrition* or over-nutrition* or overnourish* or over-nourish or adipos* or (waist adj hip adj ratio?) or (skin adj fold adj thickness) or overeat* or (over adj eat*) or ((wast* or (night adj eating)) adj (syndrome* or disorder*)) or (growth adj falter*) or ((eating or feeding or appetite or nutrition*) adj disorder?) or (weight adj length adj percentile?) or Hyperphagia* or polyphagia* or BMI or (sagittal adj abdominal adj diameter?) or (abdominal adj (fat? or (diameter adj index) or height)) or (waist adj (circumference? or (to adj (height or hip)) or height or hip)) or (lipid adj accumalat* adj product?) or hypernutrition* or hyper-nutrition*).mp.	(840722)
6	4 or 5	(995631)
7	3 and 6	(2102)
PSYCiNFO		
S5	S3 AND S4	76
S4	TI ((saudi* OR KSA OR riyadh OR Riyad OR Jedda* OR Jedde* OR “El Khobar” OR “Al Khobar” OR El-Khobar OR Al-Khobar OR Khobar OR Hijaz OR Dammam OR Medina* OR Mekka* OR Makka* OR Abha OR KAUST)) OR AB ((saudi* OR KSA OR riyadh OR Riyad OR Jedda* OR Jedde* OR “El Khobar” OR “Al Khobar” OR El-Khobar OR Al-Khobar OR Khobar OR Hijaz OR Dammam OR Medina* OR Mekka* OR Makka* OR Abha OR KAUST)) OR MW ((saudi* OR KSA OR riyadh OR Riyad OR Jedda* OR Jedde* OR “El Khobar” OR “Al Khobar” OR El-Khobar OR Al-Khobar OR Khobar OR Hijaz OR Dammam OR Medina* OR Mekka* OR Makka* OR Abha OR KAUST))	2,996
S3	S1 OR S2	98,401
S2	TI ((Obes* OR overweight* OR (over W0 (nourish* OR nutrit* OR weight*)) OR (body W0 mass W0 index*) OR (weight W0 (reduc* OR lo#s* OR decreas* OR watch* OR control*)) OR overnutrition* OR over-nutrition* OR overnourish* OR over-nourish OR adipos* OR (waist W0 hip W0 ratio#) OR (skin W0 fold W0 thickness) OR overeat* OR (over W0 eat*) OR ((wast* OR (night W0 eating)) W0 (syndrome* OR disorder*)) OR (growth W0 falter*) OR ((eating OR feeding OR appetite OR nutrition*) W0 disorder#) OR (weight W0 length W0 percentile#) OR Hyperphagia* OR polyphagia* OR BMI OR (sagittal W0 abdominal W0 diameter#) OR (abdominal W0 (fat# OR (diameter W0 index) OR height)) OR (waist W0 (circumference# OR (to W0 (height OR hip)) OR height OR hip)) OR (lipid W0 accumalat* W0 product#) OR hypernutrition* OR hyper-nutrition*)) OR AB ((Obes* OR overweight* OR (over W0 (nourish* OR nutrit* OR weight*)) OR (body W0 mass W0 index*) OR (weight W0 (reduc* OR lo#s* OR decreas* OR watch* OR control*)) OR overnutrition* OR over-nutrition* OR overnourish* OR over-nourish OR adipos* OR (waist W0 hip W0 ratio#) OR (skin W0 fold W0 thickness) OR overeat* OR (over W0 eat*) OR ((wast* OR (night W0 eating)) W0 (syndrome* OR disorder*)) OR (growth W0 falter*) OR ((eating OR feeding OR appetite OR nutrition*) W0 disorder#) OR (weight W0 length W0 percentile#) OR Hyperphagia* OR polyphagia* OR BMI OR (sagittal W0 abdominal W0 diameter#) OR (abdominal W0 (fat# OR (diameter W0 index) OR height)) OR (waist W0 (circumference# OR (to W0 (height OR hip)) OR height OR hip)) OR (lipid W0 accumalat* W0 product#) OR hypernutrition* OR hyper-nutrition*)) OR MW ((Obes* OR	95,621

	overweight* OR (over W0 (nourish* OR nutrit* OR weight*)) OR (body W0 mass W0 index*) OR (weight W0 (reduc* OR lo#s* OR decreas* OR watch* OR control*)) OR overnutrition* OR over-nutrition* OR overnourish* OR over-nourish OR adipos* OR (waist W0 hip W0 ratio#) OR (skin W0 fold W0 thickness) OR overeat* OR (over W0 eat*) OR ((wast* OR (night W0 eating)) W0 (syndrome* OR disorder*)) OR (growth W0 falter*) OR ((eating OR feeding OR appetite OR nutrition*) W0 disorder#) OR (weight W0 length W0 percentile#) OR Hyperphagia* OR polyphagia* OR BMI OR (sagittal W0 abdominal W0 diameter#) OR (abdominal W0 (fat# OR (diameter W0 index) OR height)) OR (waist W0 (circumference# OR (to W0 (height OR hip)) OR height OR hip)) OR (lipid W0 accumalat* W0 product#) OR hypernutrition* OR hyper-nutrition*))	
S1	((((((((DE "Obesity" OR DE "Overweight" OR DE "Obesity (Attitudes Toward)") OR (DE "Weight Gain")) OR (DE "Hyperphagia")) OR (DE "Eating Disorders")) OR (DE "Body Mass Index")) OR (DE "Weightlessness")) OR (DE "Feeding Disorders")) AND (DE "Eating Behavior" OR DE "Eating Disorders")	21,454
Scopus		
	(TITLE-ABS-KEY (saudi* OR ksa OR riyadh OR riyad OR jedda* OR jedde* OR "El Khobar" OR "Al Khobar" OR el-khobar OR al-khobar OR khobar OR hijaz OR dammam OR medina* OR mekka* OR makka* OR abha OR kaust)) AND (TITLE-ABS-KEY ((obes* OR overweight* OR (over PRE/0 (nourish* OR nutrit* OR weight*)) OR (body PRE/0 mass PRE/0 index*) OR (weight PRE/0 (reduc* OR lo*s* OR decreas* OR watch* OR control*)) OR overnutrition* OR over-nutrition* OR overnourish* OR over-nourish OR adipos* OR (waist PRE/0 hip PRE/0 ratio*) OR (skin PRE/0 fold PRE/0 thickness) OR overeat* OR (over PRE/0 eat*) OR ((wast* OR (night PRE/0 eating)) PRE/0 (syndrome* OR disorder*)) OR (growth PRE/0 falter*) OR ((eating OR feeding OR appetite OR nutrition*) PRE/0 disorder*) OR (weight PRE/0 length PRE/0 percentile*) OR hyperphagia* OR polyphagia* OR bmi OR (sagittal PRE/0 abdominal PRE/0 diameter*) OR (abdominal PRE/0 (fat* OR (diameter PRE/0 index) OR height)) OR (waist PRE/0 (circumference* OR (to PRE/0 (height OR hip)) OR height OR hip)) OR (lipid PRE/0 accumalat* PRE/0 product*) OR hypernutrition* OR hyper-nutrition*)))	
